# Japanese Dermatological Association Guidelines: Clinical Questions of Guidelines for Merkel Cell Carcinoma 2025

**DOI:** 10.1111/1346-8138.17974

**Published:** 2025-10-08

**Authors:** Motoki Nakamura, Kotaro Nagase, Junji Kato, Masahito Yasuda, Natsuo Tomita, Tadahiro Kobayashi, Keitaro Fukuda, Akihiko Yuki, Hiroshi Uchi, Hiroshi Koga, Tomomitsu Miyagaki, Yasuhiro Nakamura

**Affiliations:** ^1^ Department of Geriatric and Environmental Dermatology Nagoya City University Graduate School of Medical Sciences Nagoya Japan; ^2^ Nagase Dermatology Clinic Saga Japan; ^3^ Department of Dermatology Sapporo Medical University School of Medicine Sapporo Japan; ^4^ Department of Dermatology Gunma University Graduate School of Medicine Maebashi Japan; ^5^ Department of Radiology Nagoya City University Graduate School of Medical Sciences Nagoya Japan; ^6^ Department of Dermatology Public Central Hospital of Matto Ishikawa Matto Japan; ^7^ Department of Dermatology Keio University School of Medicine Tokyo Japan; ^8^ Division of Dermatology Niigata University Graduate School of Medical and Dental Sciences Niigata Japan; ^9^ Department of Dermato‐Oncology National Hospital Organization Kyushu Cancer Center Fukuoka Japan; ^10^ Kitatoda Alps Dermatology Clinic Toda Japan; ^11^ Department of Dermatology St. Marianna University School of Medicine Kawasaki Japan; ^12^ Department of Skin Oncology/Dermatology Saitama Medical University International Medical Center Hidaka Japan

**Keywords:** clinical guidelines, Japan, Merkel cell carcinoma, systematic review

## Abstract

Merkel cell carcinoma (MCC) is a highly malignant skin cancer characterized by high rates of recurrence and metastasis. Although rare, the incidence has noticeably increased in recent years. It is also known to be a highly immunogenic tumor, and the use of immune checkpoint inhibitors has begun for advanced cases. However, there had been no guidelines for this disease in Japan. Commissioned by the Japanese Dermatological Association (JDA), this revision was undertaken by a committee comprising experts across relevant fields, who meticulously reviewed and systematized a wide range of literature on MCC to create comprehensive, evidence‐based guidelines. Literature searches were conducted by the Japan Medical Library Association. The recommendation statements were determined using the GRADE Grid approach. The guidelines were developed in accordance with the “Minds Clinical Practice Guideline Creation Manual 2020 ver.3.0.” Four clinical questions (CQs) were established, and corresponding recommendation statements were provided for each. CQ1 concerns primary tumor resection margins, CQ2 concerns sentinel lymph node biopsy, CQ3 concerns postoperative radiotherapy, and CQ4 concerns chemotherapy for advanced disease. These guidelines are the first of their kind in Japan for MCC, and we hope that they will be useful not only in Japan but also in East Asia, where treatment decisions have previously had to be made based on Western guidelines.

## 1 Introduction

Merkel cell carcinoma (MCC) is a highly malignant skin cancer characterized by high rates of recurrence and metastasis. Although it is rare, there has been a noticeable increase in recent years [1, 2, 3]. It is also known to be a highly immunogenic tumor [4], and the use of immune checkpoint inhibitors has begun for advanced cases. However, due to the small number of cases, there was very little evidence available to support treatment decisions, and expert opinions were relied upon in many situations.

The main purpose of these guidelines is to provide recommendations for clinical questions related to MCC in order to improve treatment outcomes and the quality of life of patients with MCC in Japan. The committee members, who were commissioned by the Japanese Dermatological Association (JDA), reviewed the literature on MCC from various fields in an effort to create systematized guidelines. The guidelines do not impose restrictions based on sex, disease stage, severity, or comorbidities to account for the diverse patient presentations encountered in clinical settings. Intended users include MCC patients, their families, and healthcare providers involved in MCC care, encompassing dermatologists, oncologists, plastic surgeons, radiologists, nurses, pharmacists, and other allied healthcare team members, as well as policymakers. Primary care and secondary care facilities (including emergency care) are expected user environments. General clinicians are also anticipated users, with the guidelines providing practical support for effective MCC management. Especially for patients and their families, these guidelines aim to enhance understanding of MCC, fostering informed and mutually agreeable treatment decisions between healthcare providers and patients to achieve optimal healthcare outcomes.

While these guidelines represent the standard clinical approach at the time of their creation, they do not mandate specific clinical actions. Ultimately, treatment decisions should be tailored to the facility's resources (including personnel, healthcare providers' expertise, and available equipment) and the unique needs of each patient. Such decisions should be made following thorough discussions among patients, their families, and the attending physicians and other healthcare professionals involved in the care. The JDA assumes responsibility for the contents of these guidelines; however, accountability for clinical outcomes rests with the primary or attending physicians directly responsible for patient care. Neither the JDA nor the MCC Clinical Practice Guidelines Development Committee assumes responsibility for treatment outcomes. Furthermore, this document may reference drugs or dosages used in overseas clinical trials that are not yet approved in Japan.

The diagnosis and treatment of skin cancer have advanced rapidly, and treatment options have become increasingly diverse. To comprehensively incorporate these developments and ensure flexible options in clinical practice, a Skin Cancer Management Guidelines Revision Committee was established, and the MCC group was newly added as one of seven working groups (melanoma, squamous cell carcinoma, extramammary Paget's disease, basal cell carcinoma, cutaneous angiosarcoma, cutaneous lymphoma, and MCC groups). The committee included a chair, coordinating members, and representatives from each group who managed oversight within their groups and coordination across groups. Dermatologists, plastic surgeons, radiologists, oncologists, and other specialists nationwide participated as revision committee members in each group. The list of committee members involved in the MCC guidelines development is shown in Table S1. During the creation of CQs and recommendations, the committee also appointed a systematic review team to gather, evaluate, and synthesize evidence. Literature searches were conducted by expert librarians from the Japan Medical Library Association, familiar with guidelines development. The AJCC 8th Edition [5] was used for MCC staging, and the guidelines were developed in accordance with the “Minds Clinical Practice Guideline Creation Manual 2020 ver.3.0,” [6] which guided the structure and recommendation strength. Concepts widely disseminated in clinical settings and with established consensus (background questions) were summarized in the first half of the guidelines. Important clinical topics with unresolved issues were addressed in clinical questions, formulated as CQs, and discussed in the latter half of the guidelines. This English version only includes the part about CQs.

## 2 Published Work Search

Based on clinical experience and reference to previous Western guidelines, we identified key clinical issues for which sufficient consensus has not been established and formulated CQs accordingly. In setting these CQs, we examined the components of each question—Patients/Problem/Population (P), Interventions (I), Comparisons/Controls/Comparators (C), and Outcomes (O) (PICO)—and conducted a comprehensive literature search following the PICO framework. The Japan Medical Library Association was commissioned to conduct the literature search for these guidelines. For all CQs, searches were performed in The Cochrane Library, PubMed, and Ichushi‐Web from January 1972 to the fixed cutoff date of June 2023 (Table S2). Additionally, if deemed necessary by committee members responsible for the systematic review, manual searches were conducted to include reports not available in these databases or presented at major international conferences. After the literature search, each article was independently screened (second screening) for content related to key clinical issues and outcomes of benefits and harms by two reviewers: one revision committee member from the systematic review team and another from the development group who was not directly involved in the specific CQ. Based on these independent evaluations, selected studies were finalized for inclusion.

## 3 Process of Guidelines Development

### 3.1 Systematic Review Methodology

The systematic review followed the procedures outlined in the Minds Clinical Practice Guideline Development Manual 2020, ver. 3.0, utilizing the accompanying working templates provided in the manual.

First, for each CQ, the systematic review team responsible assessed individual studies grouped by outcome, examining risk of bias by study design (interventional or observational). The team evaluated various biases—including selection, performance, detection, attrition, and other potential biases—as well as issues of indirectness (such as differences in study populations, interventions, comparisons, and outcome measurements). They also extracted participant numbers. When effect measures varied across studies, these were standardized (e.g., to risk ratios or risk differences) and documented as a collective body of evidence.

The overall synthesis of the body of evidence across outcomes is referred to as the “overall synthesis of the body of evidence”, and an evaluation of the overall synthesis of the body of evidence was conducted to determine the certainty (strength) of the evidence for the overall synthesis of the body of evidence. The certainty (strength) of the evidence for the overall synthesis of the body of evidence was classified as shown in Table 1.

**TABLE 1:** Certainty (strength) of the body of evidence.

A (High): Strong confidence that the estimated effect supports the appropriateness of the recommendation.
B (Moderate): Moderate confidence that the estimated effect supports the appropriateness of the recommendation.
C (Low): Limited confidence in the appropriateness of the recommendation based on the estimated effect.
D (Very Low): Very little confidence that the estimated effect supports the appropriateness of the recommendation.

When studies shared the same design and exhibited high similarity across PICO elements, a meta‐analysis was conducted to quantitatively synthesize effect measures, incorporating this as a factor in determining the overall strength of the evidence. In cases where quantitative systematic review (meta‐analysis) was not feasible, a qualitative systematic review was performed instead. The results of these quantitative or qualitative systematic reviews were compiled into a systematic review report, representing the strength of the evidence body. This report, along with the overall summary of the evidence, served as a foundational resource for formulating recommendations.

### 3.2 Recommendation Development Methodology

Recommendations were formulated by considering both the overall certainty of evidence across outcomes and the balance between desirable effects (benefits) and undesirable effects (harms and burdens). The importance (weighting) of desirable and undesirable effects was reassessed based on the priority of each CQ and the significance of the overall evidence summary. In addition to the certainty of evidence and the balance of effects, patient values and preferences, costs, and other factors were comprehensively evaluated to determine the direction and strength of each recommendation. These considerations were discussed among CQ team members before being submitted to the recommendation decision meeting.

### 3.3 Recommendation Decision Meeting

During the Recommendation Decision Meeting (Panel Meeting) of the Revision Committee, the guidelines development team members presented their evaluation sheets, evidence summaries, and systematic review reports prepared in advance for each CQ. After these presentations, the committee members engaged in detailed discussions regarding recommendations, carefully considering the unique social context of Japan, including healthcare administration and economic factors. To incorporate diverse perspectives, patient advocacy members were also invited to participate as panelists alongside committee members and systematic review contributors. Following thorough discussion, a vote was conducted to finalize the recommendations. The voting procedures were preestablished as follows:

- As many members of the MCC guidelines development committee as possible participate in the vote.
- Members with economic conflicts of interest (COI) exceeding specified limits, academic COI, or other COIs related to the CQ under discussion refrained from voting, though they could participate in the discussions.
- Voting is conducted anonymously with the following response options:
  1. Benefit with a strong recommendation.
  2. Benefit with a weak recommendation.
  3. No benefit or risk with a weak recommendation.
  4. No benefit or risk with a strong recommendation.
  5. Unable to determine a recommendation.
- The following criteria shall be used to determine the recommended direction and strength:
  1. If 50% or more vote in favor of one direction (do/do not do) and the opposite direction receives less than 20% of the votes, the direction favored by 50% or more shall be recommended or proposed.
  2. Furthermore, if 70% or more support “strong,” it shall be considered a strong recommendation. All other cases shall be considered weak recommendations.
  3. If the above vote distribution is not achieved, further discussion will be conducted, and a re‐vote will be implemented. If no consensus is reached after two votes, no recommendation (unable to determine recommendation) will be made.

Prior to each CQ vote, the presence of various COIs was reconfirmed, and committee members with COIs exceeding the specified limits were required to abstain from voting. However, no committee members had COIs in any of the CQs, and no members abstained from voting. The results of the vote were included in the explanatory notes for each CQ.

All “Recommendation Development‐Related Materials” used in the decision‐making process were published on the JDA website's “Guidelines and Protocols” page, together with these guidelines.

No specific changes were made to the CQ numbers during the drafting process.

The first edition of the MCC Guidelines commenced its development process in March 2023, with the first Committee meeting. Subsequently, these guidelines were developed through the following steps: two Committee meetings, five MCC Guideline Group meetings, multiple rounds of email consultations, three rounds of public comments, and an external evaluation through expert feedback.

## 4 External Review, Public Comment, and Expert Feedback

The revised guidelines were evaluated by three external review groups. One review was conducted by council members of the JDA who were not part of the guideline development committee (October 23–November 21, 2024). The Japanese Skin Cancer Society also provided expert commentary from council members (October 11–November 8, 2024). Additionally, public comments were solicited through the EBM Promotion Project Minds, managed by the Japan Council for Quality Health Care, and the Japanese Skin Cancer Society's website (October 14–November 11, 2024). Based on the feedback from these public and expert comments, adjustments were made to finalize the recommendations.

## 5 Future Revisions and Goals for Guidelines Development

With continued advancements in medical science and societal shifts, it is anticipated that the clinical approach to MCC will also undergo significant changes. Therefore, periodic revisions of these guidelines are necessary. Following the established protocol, a revision will be conducted approximately every 3–4 years, with interim revisions made as needed, and updates will be published on the JDA's website.

## 6 Post‐Publication Monitoring of the Guidelines

Following the release of the guidelines, a survey will be conducted to assess its dissemination and any changes in clinical practice.

## 7 CQ1: Is a Lateral Margin of ≤ 1 cm Recommended for Surgical Treatment of the Primary Lesion of Merkel Cell Carcinoma?

### 7.1 Recommendation

If postoperative radiotherapy is not performed, excision with a lateral margin of ≤ 1 cm is not recommended.

If postoperative radiotherapy is performed, excision with a lateral margin of ≤ 1 cm is not suggested.

When postoperative radiotherapy is not performed.

Recommendation: 4.

Level of evidence: D.

Agreement rate: 100% (8/8).

When postoperative radiotherapy is performed.

Recommendation: 3.

Level of evidence: D.

Agreement rate: 100% (8/8).

### 7.2 Voting Results

Without postoperative radiotherapy.

**Table:** 

	1. Benefit with a strong recommendation	2. Benefit with a weak recommendation	3. No benefit or risk with a weak recommendation	4. No benefit or risk with a strong recommendation	5. Unable to determine recommendation
CQ (1st vote)				100% (8/8)	
Total votes: 8 (abstention: 0)					

With postoperative radiotherapy.

**Table:** 

	1. Benefit with a strong recommendation	2. Benefit with a weak recommendation	3. No benefit or risk with a weak recommendation	4. No benefit or risk with a strong recommendation	5. Unable to determine recommendation
CQ (1st vote)			100% (8/8)		
Total votes: 8 (abstention: 0)					

### 7.3 Background and Purpose

MCC is a highly malignant tumor. According to the NCCN guidelines (version 1, 2024), wide local excision is recommended for the primary lesion [7]. However, the lateral margin is not clearly defined, ranging between 1 and 2 cm. The guidelines further recommend the use of postoperative radiotherapy for patients with specific risk factors, including: (1) a primary lesion exceeding 1 cm, (2) primary lesions located on the head and neck, (3) lymphatic infiltration, (4) patients with organ transplants, (5) HIV‐positive patients, (6) patients with chronic lymphocytic leukemia, and (7) patients with chronically suppressed T‐cell immunity. Given that MCC commonly occurs in the head and neck region, the majority of cases in clinical practice receive postoperative radiotherapy. It is crucial to establish an appropriate margin, considering the outcomes of cases with and without adjuvant therapy. This clarity is essential for determining the optimal treatment strategy, which led to the formulation of this CQ.

### 7.4 Scientific Evidence

This study focused on patients (P) with MCC presenting with a primary lesion but without distant metastasis. The intervention (I) was defined as surgical excision with a margin of ≤ 1 cm, while the comparison (C) was excision with a margin of ≥ 1 cm. The primary outcomes (O) were defined as recurrence‐free survival, local recurrence rate, overall survival (OS), and positive margins. Four studies, all of which were retrospective cohort studies, were included in the systematic review.

For recurrence‐free survival, only one study was included in the systematic review, and it showed no significant difference between excision with a margin ≤ 1 cm and margin > 1 cm (hazard ratio [HR], 1.10; 95% confidence interval [CI], 0.60–2.02; *p* = 0.74) [8]. However, the proportion of cases receiving postoperative radiotherapy was high in both groups (82.8% in the margin ≤ 1 cm group and 77.6% in the margin > 1 cm group).

Regarding the positive resection margin rate, three studies were included in the systematic review [8, 9, 10]. In each of these studies, most cases received postoperative radiotherapy. A pooled analysis of three studies demonstrated that the positive resection margin rate was significantly higher for excisions with a margin of ≤ 1 cm than for those with a margin > 1 cm (relative risk, 3.40; 95% CI, 2.89–4.02; *p* < 0.001).

For local recurrence rate, three studies were included in the systematic review [8, 9, 10, 11]. In one of these studies, 69% of patients received postoperative radiotherapy [10]. However, this study analyzed local recurrence rates without distinguishing between cases with and without postoperative radiotherapy and found no significant difference between excision with a margin of ≤ 1 cm and margin > 1 cm (*p* = 0.67). The other two studies performed separate analyses based on the presence or absence of postoperative radiotherapy [8, 11]. A pooled analysis of these two studies indicated that the local recurrence rate for excision with a margin of ≤ 1 cm was significantly higher than that for a margin > 1 cm (*p* = 0.007). Conversely, no significant difference was observed between cases with a margin of ≤ 1 cm plus postoperative radiotherapy and those with a margin of > 1 cm plus postoperative radiotherapy (relative risk, 0.62; 95% CI, 0.14–2.73; *p* = 0.56) [8, 11].

In OS, three studies were included in the systematic review [8, 9, 10]. In two of these studies, most patients received postoperative radiotherapy. However, OS was analyzed without separating cases based on the use of postoperative radiotherapy, and no significant difference in OS was observed between those with a margin ≤ 1 cm and a margin > 1 cm [8, 10].

The other study included 6156 patients and performed an analysis stratified using postoperative radiotherapy [9]. The results were as follows:

Compared to those with a margin of ≤ 1 cm: (1) a margin of ≤ 1 cm plus postoperative radiotherapy was associated with significantly improved OS (HR, 0.81; 95% CI, 0.74–0.89; *p* < 0.001); (2) a margin of > 1 cm alone showed significantly improved OS (HR, 0.80; 95% CI, 0.71–0.89; *p* < 0.001); (3) a margin of > 1 cm plus postoperative radiotherapy showed the most substantial OS benefit (HR, 0.59; 95% CI, 0.52–0.65; *p* < 0.001) [9]. Furthermore, in multivariate analysis, patients with a margin of > 1 cm plus postoperative radiotherapy had significantly improved OS compared to those with a margin of ≤ 1 cm plus postoperative radiotherapy (HR, 0.87; 95% CI, 0.76–0.98; *p* = 0.03) [9]. Although there was no significant difference in OS between groups with margin ≤ 1 cm plus postoperative radiotherapy and margin > 1 cm (*p* = 0.87), postoperative radiotherapy was associated with a significant OS benefit, regardless of margin size (HR, 0.78; 95% CI, 0.72–0.84; *p* < 0.001) [9].

### 7.5 Comments

In clinical practice, postoperative radiotherapy is frequently used, and this trend was also observed in the four studies included in the systematic review, where 70%–80% of cases received postoperative radiotherapy. However, one of the four studies analyzing recurrence‐free survival, one of three studies assessing local recurrence rates, and two of three studies evaluating OS did not differentiate cases based on the use of postoperative radiotherapy. Thus, this approach introduced a bias regarding the impact of radiotherapy on prognosis. As a result, no significant differences in recurrence‐free survival, local recurrence rates, or OS were observed between groups with a margin of ≤ 1 cm and > 1 cm.

On the other hand, in studies that compared prognosis based on the presence or absence of postoperative radiotherapy, the local recurrence rate was higher in cases with a margin of ≤ 1 cm than in those with > 1 cm. However, no significant difference was found between cases with a margin of ≤ 1 cm plus postoperative radiotherapy and those with a margin of > 1 cm. These results suggest that postoperative radiotherapy contributes to reducing local recurrence rates. In terms of OS, significant improvements were observed in the following scenarios compared to a margin of ≤ 1 cm alone: (1) a margin ≤ 1 cm plus postoperative radiotherapy, (2) a margin > 1 cm, and (3) a margin > 1 cm plus postoperative radiotherapy. Furthermore, OS was significantly improved in cases with a margin > 1 cm plus postoperative radiotherapy compared to those with a margin ≤ 1 cm plus postoperative radiotherapy (HR, 0.87; 95% CI, 0.76–0.98; *p* = 0.03). However, there was no significant difference in OS between a margin ≤ 1 cm plus postoperative radiotherapy and a margin > 1 cm [9]. These findings indicate that postoperative radiotherapy improves OS regardless of margin size.

During the panel meeting, it was noted that all studies included in the systematic review were retrospective cohort studies. The panel raised concerns about potential selection bias, where cases with a larger margin might involve tumors that were easier to excise, potentially leading to better baseline prognosis. Consequently, the certainty of the evidence was deemed very low. The limited evidence also made it difficult to evaluate the balance between benefits and harms. In addition, 70%–80% of cases in all four studies received postoperative radiotherapy. Studies that compared prognosis based on the presence or absence of postoperative radiotherapy consistently showed that the use of radiotherapy was associated with lower local recurrence rates and longer OS. Given this, the panel considered studies that did not differentiate between cases with and without postoperative radiotherapy to have analyzed outcomes with a bias introduced by the impact of radiotherapy on prognosis. Based on this understanding, the panel assessed treatment outcomes and made conclusions.

- Without postoperative radiotherapy: The panel strongly recommends against excision with a margin of ≤ 1 cm due to higher local recurrence rates and worse OS.
- With postoperative radiotherapy: Although there was no significant difference in OS between a margin of ≤ 1 cm plus postoperative radiotherapy and a margin of > 1 cm, OS was significantly improved with a margin of > 1 cm plus postoperative radiotherapy. As a result, the panel weakly recommended against excision with a margin of ≤ 1 cm, even with postoperative radiotherapy.

### 7.6 Salient Aspects for Clinical Application

Resection with a margin > 1 cm may be difficult depending on the site and size of the tumor. In such cases, the use of postoperative radiotherapy should be considered. Postoperative radiotherapy requires regular hospital visits or hospitalization for a certain period. Therefore, when resection with a margin > 1 cm is feasible, treatment decisions should consider not only the condition of the MCC but also the patient's social circumstances (such as whether the patient can maintain regular visits for the radiotherapy). The options include: (1) excision with a margin > 1 cm alone, (2) prioritizing margin preservation by selecting a margin of ≤ 1 cm plus postoperative radiotherapy, or (3) prioritizing improved OS with a margin of > 1 cm plus postoperative radiotherapy. In addition, delaying the start of postoperative radiotherapy beyond 8 weeks after surgery has been associated with increased risks of recurrence and disease‐specific mortality [12]. If a margin of > 1 cm necessitates complex reconstruction, which may delay wound healing and the initiation of radiotherapy, an excision with a margin of ≤ 1 cm should be considered.

### 7.7 Study Subjects in the Future

Due to the rarity of MCC, no prospective clinical trials have been reported to date focusing on the surgical margins for the primary lesion of MCC. It is difficult to conduct randomized controlled trials. As an alternative, a prospective single‐arm study with historical controls is warranted, evaluating recurrence‐free survival, OS, and local recurrence rates for excision with a margin of ≤ 1 cm. In cases where MCC is difficult to excise, radiotherapy alone is sometimes used. Therefore, studies comparing the prognosis of radiation alone, margin ≤ 1 cm ± postoperative radiotherapy, and margin > 1 cm ± postoperative radiotherapy after adjusting for background factors are also desirable.

## 8 CQ2 Is Sentinel Node Biopsy Recommended for Merkel Cell Carcinoma?

### 8.1 Recommendation

Sentinel node biopsy is suggested for Merkel cell carcinoma.

Recommendation: 2.

Level of evidence: D.

Agreement rate: 100% (8/8).

### 8.2 Voting Results

**Table:** 

	1. Benefit with a strong recommendation	2. Benefit with a weak recommendation	3. No benefit or risk with a weak recommendation	4. No benefit or risk with a strong recommendation	5. Unable to determine recommendation
CQ (1st vote)		100% (8/8)			
Total votes: 8 (abstention: 0)					

### 8.3 Background and Purpose

The majority (79%) of first metastases of MCC occur in regional lymph nodes [13]. The rate of regional lymph node metastasis is reported to be 14% even when the largest diameter of the primary tumor is 0.5 cm, and 25% when the largest diameter is 1.7 cm. Even if the primary tumor is small, there is a risk of lymph node metastasis [14]. In addition, the positive rate of sentinel lymph node (SLN) metastasis of MCC is approximately 30%, which is higher than that observed in melanoma [15, 16, 17, 18]. This suggests that subclinical metastasis may already be present, even if the lymph nodes are not clinically enlarged. Sentinel lymph node biopsy (SLNB) for MCC was approved for insurance coverage in 2018; however, the implementation rate was only 35.9% for Stage I and 46.6% for Stage II in the United States [19], and similarly low rates are expected in Japan [19]. Since this syndrome is more prevalent in the head and neck region of elderly patients, it is assumed that the low rate of SLNB is due to the fact that invasive SLNB may be difficult to perform due to the decreased performance status and comorbidities associated with advanced age, and that several physicians may be unfamiliar with SLNB of the neck region and may omit SLNB. It is necessary to clarify whether or not SLNB improves prognosis, and to select appropriate treatment based on scientific evidence, regardless of the above factors.

### 8.4 Scientific Evidence

Patients with MCC without clinically evident regional lymph node metastases were included. In this context, the Patient (P) and Intervention (I) were defined as those undergoing SLNB, while the Comparison (C) group comprised patients who did not undergo SLNB. The primary Outcomes (O) assessed were OS, recurrence‐free survival, treatment‐related adverse events, and cost.

There were no randomized controlled trials or prospective studies investigating SLNB in MCC. Similarly, no studies in the Japanese literature examined the prognostic value of SLNB. This review included four retrospective studies in which SLNB was performed and sufficient prognostic data were provided [18, 20, 21, 22].

In a retrospective study by Sattler et al. [22] reported in 2013, OS was significantly longer in the SLNB group (*n* = 19) than in the non‐SLNB group (*n* = 28) (mean survival: 211 months vs. 72 months; *p* = 0.008). Similarly, a large retrospective study by Xia et al. [21] in 2020 found that patients in the SLNB group (*n* = 690) had significantly longer OS than the those in the non‐SLNB group (*n* = 786) (univariate analysis: HR, 0.382; *p* < 0.001; multivariate analysis: HR, 0.327; *p* = 0.005). In a retrospective study by Moon et al. [20] in Korea, reported in 2023, there was a trend toward longer OS in the SLNB group (*n* = 8) compared to the no SLNB group (*n* = 42); however, the difference was not significant (*p* = 0.066). The lack of significance was attributed to the small sample size and limited number of events in both groups.

MCC predominantly affects elderly individuals, and as a result, non‐MCC‐related deaths are relatively common. Although disease‐specific survival (DSS) was not included as an outcome in this clinical question (CQ), we also examined DSS as a supplementary endpoint. DSS was evaluated in two studies. In the study by Xia et al. [21], both univariate and multivariate analyses of DSS demonstrated that SLNB was associated with significantly longer DSS compared to no SLNB (univariate analysis: HR, 0.412; *p* < 0.001; multivariate analysis: HR, 0.089; *p* = 0.004). In a retrospective study by Kachare et al. [18], DSS was significantly longer in the SLNB‐treated group (*n* = 474) than in the non‐treated group (*n* = 719) (5‐year DSS: 79.2% vs. 73.8%; *p* = 0.004). However, whether the improved DSS was attributable to SLNB itself or to posttreatment selection bias was not examined [18].

Sattler et al. [22] reported a trend toward increased disease‐free survival in the SLNB group than in the non‐SLNB group, but the difference was not significant (mean disease‐free survival: 147 months vs. 61 months; *p* = 0.173). In a retrospective study by Moon et al. [20], the SLNB group had significantly longer recurrence‐free survival than the non‐SLNB group (mean recurrence‐free survival: 62.57 months vs. 24.82 months; *p* = 0.036).

No studies examined treatment‐related adverse events or costs.

### 8.5 Comments

All four studies included in the systematic review were retrospective observational studies, and there were differences among the background factors for the presence or absence of SLNB, with the non‐SLNB group including more cases in poor general health, more primary head and neck cases, and more elderly patients, suggesting that these factors may be at risk of bias affecting the outcomes. This may be a risk of bias. In addition, whether posttreatment after SLNB had an effect on prognosis was not examined. In addition, only one of the four studies by Xia et al. [21] adjusted for confounding factors. Therefore, the evidence to positively recommend SLNB for MCC is weak. Despite such weak evidence, a study with a large sample size showed that both OS and DSS were prolonged in the SLNB group, and multivariate analysis also showed that SLNB had an impact on the improvement of OS and DSS. These points were thoroughly discussed at the panel meeting before the vote, and the result was “Sentinel lymph node biopsy is suggested for MCC.”

### 8.6 Salient Aspects for Clinical Application

Although SLNB has an impact on OS improvement, it is unclear whether SLNB itself or the choice of posttreatment has an impact on prognosis. On the other hand, there are cases in which SLNB is practically difficult to perform because of the prevalence of elderly patients, and cervical SLNB may affect the SLN identification rate and diagnostic accuracy when performed by inexperienced physicians.

### 8.7 Study Subjects in the Future

Randomized controlled trials comparing two groups, SLNB and no SLNB, are difficult to conduct because of the rarity of this disease, and it is necessary to compare various outcomes in a prospective single‐arm validation study with a historical control adjusted for patient background.

## 9 CQ3‐1 Is Postoperative Radiotherapy Recommended for Primary Merkel Cell Carcinoma When Resection Margins Are Negative?

### 9.1 Recommendation

We suggest postoperative radiotherapy for primary Merkel cell carcinoma when resection margins are negative.

Recommendation: 2.

Level of evidence: D.

Agreement rate: 100% (8/8).

### 9.2 Voting Results

**Table:** 

	1. Benefit with a strong recommendation	2. Benefit with a weak recommendation	3. No benefit or risk with a weak recommendation	4. No benefit or risk with a strong recommendation	5. Unable to determine recommendation
CQ (1st vote)		100% (8/8)			
Total votes: 8 (abstention: 0)					

### 9.3 Background and Purpose

MCC is generally considered high‐grade, and a high local recurrence rate has been reported with resection of the primary tumor alone. Although there are no randomized controlled trials evaluating the significance of postoperative radiotherapy, there are many retrospective studies that suggest its efficacy. However, some previous studies were unclear about whether postoperative irradiation was limited to the primary tumor, the primary + regional lymph node group, or an apparent mixture of the two, making it difficult to interpret outcomes regarding the benefit of postoperative radiotherapy. Late effects of radiotherapy are also an important concern because of the predilection of MCC for the head and neck region. Therefore, we will discuss the significance of postoperative radiotherapy to the primary tumor in patients with negative margins after resection of the primary tumor.

### 9.4 Scientific Evidence

Patients (P) with resected primary lesions and negative margins of MCC were evaluated for outcomes with intervention (I) as postoperative radiotherapy to the primary lesion. The comparison (C) was no postoperative radiotherapy. The primary outcomes (O) included OS, recurrence‐free survival, local recurrence rate, and adverse events.

There were no interventional studies such as randomized controlled trials or prospective observational studies related to this CQ. Although there are many retrospective studies analyzing OS, including those using large data sets such as the US Cancer Registry database and the Surveillance Epidemiology and End Results (SEER) database, many of these studies did not provide details of the irradiation field, and we judged them to be inappropriate for use in the evaluation of this CQ [23, 24, 25]. However, the details of the irradiation field are unknown in many studies, and these studies were judged to be inappropriate for use in the evaluation of this CQ [23, 24, 25]. In addition, there were no reports of postoperative radiotherapy specifically for the cases with negative margins after resection of the primary tumor, so we adopted the studies in which postoperative radiotherapy was given regardless of the presence or absence of margins as alternatives. Three studies were finally adopted for the systematic review [11, 26, 27].

In a single center retrospective study of Stages I–II MCC by Kang et al. [26], recurrence‐free survival was significantly longer in 32 patients in the intervention group (postoperative radiotherapy) than in 10 patients in the control group (no postoperative radiotherapy) (2‐year recurrence‐free survival: 89% vs. 36%; *p* < 0.001). However, the sample size was small and there was no clear description of the status of the transection [26].

In a single center retrospective study by Tarabadkar et al. [11], the local recurrence rate was significantly higher in the intervention group (140 cases) compared to the control group (48 cases) (4‐year local recurrence rate 1% vs. 15%; *p* = 0.001). However, there was no clear description of the status of the disconnection in this study, and the intervention group had fewer head and neck primary sites than the control group (26% vs. 63%), and there was variation in patient background.

Adverse events have been evaluated in two studies: a study by Takagishi et al. [27] comparing 23 patients in the intervention group with 23 patients in the control group in Stage I with a head and neck primary, and a study by Kang et al. [26] comparing 43 patients in the intervention group and 10 patients in the control group in Stages I–II, which includes other areas than the head and neck as primary. Both groups reported no serious adverse events. However, the sample size was small, and the dose, irradiation site, type of radiation, and definition of late adverse events were not standardized.

There was no literature suitable for this CQ evaluation regarding OS.

### 9.5 Comments

A systematic review suggested that postoperative radiotherapy to the primary tumor after resection of the primary MCC prolonged recurrence‐free survival and reduced local recurrence, but did not improve OS. The effect of postoperative radiotherapy on adverse events was inconclusive.

Many of the panelists agreed that postoperative radiotherapy is likely to be a superior intervention, as it has been reported to reduce local recurrence in many cases. On the other hand, it was also discussed that OS could not be evaluated and that it is difficult to properly assess the balance of benefit and harm because the studies used in this study were all small sample size, retrospective, observational studies. It was also discussed that the evidence used in this CQ was not based on studies in which postoperative radiotherapy was given only to patients with negative margins after resection of the primary tumor. After these discussions, the vote was limited to “weakly recommend intervention.”

### 9.6 Salient Aspects for Clinical Application

Because the effectiveness of postoperative radiotherapy on OS is unknown, patients with a low probability of local recurrence after resection of the primary tumor should consider not receiving postoperative radiotherapy. Postoperative radiotherapy requires a certain period of either inpatient or outpatient care. The appropriateness of intervention should be decided based on the patient's social circumstances (e.g., elderly patients who have difficulty making daily visits to the hospital) and desires.

### 9.7 Study Subjects in the Future

Several cohort studies have shown that postoperative radiotherapy was associated with fewer recurrences or longer OS compared to surgical therapy alone. A large study using the US Cancer Registry database, which included of 4843 [23, 24] and 1795 [25] patients with Stages I–II disease, concluded that OS was significantly longer than with surgery alone [23, 24, 25]. However, these studies were not adopted in this systematic review because the irradiation fields were unclear. In the future, it is expected that more precise recommendations will become possible by accumulating reports of larger studies in which the irradiation field is limited to the primary lesion only, and by conducting a systematic review on such reports.

Although the incidence of MCC caused by UV exposure and Merkel cell polyomavirus (MCPyV) differs among races, there are no studies on the benefit of adjuvant radiotherapy to primary tumors in Asian patients. Future studies are expected to accumulate data that include more Asian patients and factors such as MCPyV infection to investigate the usefulness of adjuvant radiotherapy to primary tumors.

## 10 CQ3‐2 if Sentinel Lymph Node Metastasis Was Negative, Is Postoperative Radiotherapy for the Regional Lymph Node Recommended?

### 10.1 Recommendation

We suggest not performing postoperative radiotherapy to regional lymph nodes if sentinel lymph node metastasis was negative.

Recommendation: 3.

Level of evidence: D.

Agreement rate: 75.0% (6/8).

### 10.2 Voting Results

**Table:** 

	1. Benefit with a strong recommendation	2. Benefit with a weak recommendation	3. No benefit or risk with a weak recommendation	4. No benefit or risk with a strong recommendation	5. Unable to determine recommendation
CQ (1st vote)				75.0% (6/8)	25.0% (2/8)
Total votes: 8 (abstention: 0)					

### 10.3 Background and Purpose

MCC is generally considered a high‐grade tumor with a high SLN metastasis rate of approximately 30%, and numerous studies have reported the efficacy of postoperative radiotherapy for regional lymph node groups. However, the presence or absence of regional lymph node metastases, the size and number of lymph nodes involved, and other factors vary with the progression of the disease among patients. Because MCC is predominantly located in the head and neck region, adverse events associated with radiotherapy in this region are an important cosmetic and functional concern. Therefore, it remains unclear which patients would benefit most from postoperative radiotherapy in terms of the balance of benefits and harms. Furthermore, false‐negative SLN metastases are significantly associated with worse survival compared to true negative SLN metastases (5‐year disease‐specific survival: 51.3% vs. 90.9%; *p* < 0.001) [28], which may clarify the need for postoperative radiotherapy to regional lymph nodes in SLN‐negative cases and help to better select patients in need. Therefore, it is necessary to clarify the necessity of postoperative radiotherapy for regional lymph node groups in SLN‐negative patients and to select patients who need it more appropriately.

### 10.4 Scientific Evidence

Patients (P) with SLN‐negative MCC were evaluated for outcomes with intervention (I) as postoperative radiotherapy to the regional lymph node group. The comparison (C) was no postoperative radiotherapy to the regional lymph node group. The primary outcome (O) was OS and local recurrence rate.

There were no interventional studies such as randomized controlled trials or prospective observational studies related to this CQ. Although retrospective studies using large‐scale data such as the US Cancer Registry database and the Surveillance, Epidemiology, and End Results (SEER) database analyzed OS, the details of the irradiation field were not known, and we judged that it was inappropriate to use these data to evaluate this CQ [23, 24, 25]. In addition, because there were no reports of postoperative radiotherapy specifically for SLN‐negative cases, we adopted as an alternative a study in which postoperative radiotherapy was given to patients who had lymph node dissection after positive sentinel lymph node biopsy results. As a result, two studies were finally adopted for the systematic review [29, 30].

Local recurrence (within the regional lymph node group) has been evaluated in two studies: a subgroup analysis comparing 10 patients in the intervention group (postoperative radiotherapy) to 20 patients in the control group (no postoperative radiotherapy) in a retrospective observational study by Dinges et al. and a retrospective observational study comparing 14 patients in the intervention group to 97 patients in the control group by Grotz et al. study [29, 30]. Both studies found no significant difference in recurrence rates between the intervention and control groups (10% vs. 20%; *p* = 0.64; 7% vs. 14.4%; *p* = 0.45).

OS has been evaluated in one study, and the aforementioned study by Dinges et al. [30] compared 10 patients in the intervention group with 20 patients in the control group and found no significant difference (1‐year survival: 100% vs. 95%; 5‐year survival: 80% vs. 73.7%; *p* = 0.65).

There were no reports of recurrence‐free survival, posttreatment adverse events, or cost for the systematic review.

### 10.5 Comments

The review of studies with strictly defined irradiation fields showed no statistically significant differences in local recurrence or OS between the intervention and control groups, suggesting no benefit from postoperative radiotherapy. The panel also considered the use of postoperative radiotherapy to regional lymph nodes in SLN‐negative patients to be even less meaningful because these studies were not specific to SLN‐negative patients, but rather to a small number of patients who had SLN‐positive disease and underwent dissection. Furthermore, the panel felt that it was difficult to properly assess the balance of benefit and harm because of the different study populations, and that no definitive conclusions could be drawn. After these discussions, the vote was to recommend no postoperative radiotherapy, with a few votes for “unable to determine recommendation”.

### 10.6 Salient Aspects for Clinical Application

It is important to note that none of the studies used in this CQ were specific to SLN‐negative cases. In addition, the majority of the data presented in this CQ were from patients without clinically evident regional lymph node metastases who underwent dissection due to pathologically identified micrometastases. In practice, there are cases with clinical metastases that are dissected, and the benefit of radiotherapy in such cases is currently unclear.

### 10.7 Study Subjects in the Future

Since there have been reports of false‐negative SLN metastases in this disease, the NCCN guidelines (version 1, 2024) suggest that postoperative radiotherapy to regional lymph node groups be considered for SLN‐negative cases in patients at high risk for false‐negative results, such as those with abnormal lymph flow or multiple SLNs, and in patients with severe immunosuppression [7]. The German S2k guidelines consider radiotherapy to the regional lymph node group in SLNB‐negative patients with T2 or higher and head and neck patients with complicated lymphatic flow, while other patients should be followed up [31]. In the future, more accurate evaluation and precise recommendations will become possible with the accumulation of prospective studies with more clearly defined irradiation fields and systematic reviews.

## 11 CQ3‐3 Is Postoperative Radiotherapy for Regional Area Recommended After Regional Lymph Node Dissection?

### 11.1 Recommendation

We suggest that postoperative radiotherapy not be administered for the regional area after regional lymph node dissection.

Recommendation: 3.

Level of evidence: D.

Agreement rate: 87.5% (7/8).

### 11.2 Voting Results

**Table:** 

	1. Benefit with a strong recommendation	2. Benefit with a weak recommendation	3. No benefit or risk with a weak recommendation	4. No benefit or risk with a strong recommendation	5. Unable to determine recommendation
CQ (1st vote)			87.5% (7/8)		12.5% (1/8)
Total votes: 8 (abstention: 0)					

### 11.3 Background and Purpose

MCC is generally considered a high‐grade tumor, and numerous studies have reported the efficacy of postoperative radiotherapy for regional lymph nodes. However, the progression of the disease varies among cases, depending on the presence or absence of regional lymph node metastases, as well as the size and number of lymph nodes involved. Because MCC is predominantly located in the head and neck region, adverse events associated with radiotherapy in this region are an important cosmetic and functional concern. Therefore, it remains unclear in which patients benefit most from postoperative radiotherapy in terms of the balance of benefits and harms. More accurate selection is needed for patients who require postoperative radiotherapy to regional lymph node groups.

### 11.4 Scientific Evidence

Patients (P) with MCC who underwent regional lymph node dissection were evaluated for outcomes with intervention (I) as postoperative radiotherapy for the regional lymph node group. The comparison (C) was no postoperative radiotherapy to the regional lymph node group. The primary outcomes (O) were OS, recurrence‐free survival, and local recurrence rate.

No interventional studies such as randomized controlled trials or prospective observational studies were found to be relevant to this CQ. Although there are many retrospective studies analyzing OS, including those using large data sets such as the US Cancer Registry database and the SEER database, many studies did not specify whether the irradiation field was limited to the primary tumor or included regional lymph nodes. Many of these reports were judged to be inappropriate for use in the evaluation of this CQ [23, 24, 25]. As a result, three studies were finally included in the systematic review [32, 33, 34].

OS was evaluated in two studies. In a study by Perez et al. [33] comparing three groups: 11 patients in the intervention (postoperative radiotherapy to regional lymph nodes) group and 20 patients in the control (no postoperative radiotherapy) group, plus 41 patients who received radiotherapy without regional lymph node dissection (radiation alone), no significant difference was found among the three groups (3‐year survival: 69% vs. 71% vs. 67%; *p* = 0.72). In a retrospective study by Ma et al. [32] comparing 183 patients in the intervention group and 606 patients in the control group, the two groups were adjusted for background factors by inverse probability weighting, which also showed no significant difference (5‐year survival rate: 46.3% vs. 46.6%; *p* = 0.99).

A single center study by Andruska et al. [34] revealed that recurrence‐free survival was significantly longer in 18 patients in the intervention group than in 32 in the control group by multivariate analysis (HR, 0.09; 95% CI, 0.02–0.33; *p* < 0.001).

Local recurrence has been evaluated in two studies. Andruska et al. [34] found that the intervention significantly reduced local recurrence by multivariate analysis using the Cox proportional hazards model (HR, 0.04; 95% CI, 0.01–0.37; *p* = 0.004). On the other hand, Perez et al. [33] compared local recurrence‐free survival in intervention, control, and radiation alone groups and found no significant difference in among the three groups (2‐year local recurrence‐free rate: 94% vs. 100% vs. 93%; *p* = 0.60).

There were no studies on posttreatment complications or costs.

### 11.5 Comments

A systematic review found no statistically significant difference in OS between the intervention and control groups, suggesting no benefit from postoperative radiotherapy to the regional lymph node group. On the other hand, a possible improvement in recurrence‐free survival was suggested in the study by Andruska et al. [34]. No consistent results were obtained for local recurrence. In addition, all studies had small samples, and although some studies performed multivariate analysis, there was an imbalance in patient background, with more head and neck sites in the intervention group (percentage of head and neck sites in the intervention and control groups: 69% vs. 22% in the Andruska et al. study and 50.0% vs. 27.3% in the Perez et al. study), suggesting that the intervention group was more likely to have head and neck tumors than the control group [33, 34]. It was argued that it is difficult to properly assess the balance between benefit and harm. Therefore, many of the panel members felt that postoperative radiotherapy to the regional lymph node group was not worthwhile after regional lymph node dissection, with a few voting “unable to determine recommendation”, but with a weak recommendation not to intervene.

### 11.6 Salient Aspects for Clinical Application

Although there was no evidence of an OS benefit, there were reports of improvement in recurrence‐free survival and local recurrence, suggesting that postoperative radiotherapy may be considered in cases where regional lymph node dissection was not sufficiently performed and local recurrence was highly likely. On the other hand, postoperative radiotherapy requires a certain period of either inpatient or outpatient care. The appropriateness of intervention should be decided based on the patient's social circumstances (e.g., elderly patients who have difficulty making daily visits to the hospital) and desires.

### 11.7 Study Subjects in the Future

This CQ is evaluating whether to add radiotherapy after lymph node dissection, and the discussion is based on the premise that lymph node dissection has been performed. In 2019, the most frequently selected treatments were lymph node dissection, radiation alone, and lymph node dissection followed by postoperative radiotherapy, with the proportion of radiotherapy alone increasing each year [32]. In this study, there was no significant difference in OS between lymph node dissection and radiation alone. In addition, a prospective study of SLN‐positive cases by Lee et al. [35] found no significant difference in either OS or disease‐free survival between lymph node dissection and radiotherapy alone. Since 2008, intensity‐modulated radiotherapy has been covered by insurance in Japan, leading to improvements in the precision and effectiveness of radiation delivery. In the future, it is necessary to accumulate evidence for optimal treatment selection, including radiotherapy alone, for patients with regional lymph node metastases.

## 12 CQ4 Is Systemic Chemotherapy Other Than EP/EC Recommended for Advanced Merkel Cell Carcinoma That Is Failure or Unsuitable for Immunotherapy?

### 12.1 Recommendation

No clear recommendation can be made for any systemic chemotherapy for advanced Merkel cell carcinoma that is failed or unsuitable for immunotherapy.

Recommendation: 5.

Level of evidence: D.

Agreement rate: 87.5% (7/8).

### 12.2 Vote Results

**Table:** 

	1. Benefit with a strong recommendation	2. Benefit with a weak recommendation	3. No benefit or risk with a weak recommendation	4. No benefit or risk with a strong recommendation	5. Unable to determine recommendation
CQ (1st vote)				12.5% (1/8)	87.5% (7/8)
Total votes: 8 (abstention: 0)					
CQ (2nd vote)				12.5% (1/8)	87.5% (7/8)
Total votes: 8 (abstention: 0)					

### 12.3 Background and Purpose

There are no cytotoxic anticancer drugs covered by insurance in Japan for advanced MCC, and chemotherapy such as etoposide + cisplatin/etoposide + carboplatin (EP/EC therapy) has been tried in accordance with small cell lung cancer. Currently, since avelumab, an anti‐PD‐L1 antibody, was covered by insurance in 2017, there is no dispute that avelumab is the first choice from the viewpoint of insurance coverage. If tumor mutational burden‐high is identified by oncogene panel testing, pembrolizumab, an anti‐PD‐1 antibody, is also an option. However, the benefit of chemotherapy with cytotoxic agents for patients who are refractory or unsuitable for immunotherapy is unknown. Therefore, we established this CQ to clarify whether systemic chemotherapy other than EP/EC is effective in immunotherapy‐refractory or ‐refractory patients, assuming that EP/EC is the second‐line therapy (deemed standard therapy).

### 12.4 Scientific Evidence

Patients (P) with unresectable MCC refractory or unsuitable for immunotherapy were treated with systemic chemotherapy other than EP/EC as intervention (I), and outcomes were evaluated for each treatment modality. The comparison (C) was EP/EC. The drugs used in the intervention were limited to those available in Japan. The primary outcomes (O) were overall response rate (ORR), duration of response (DOR), OS, progression‐free survival (PFS), and adverse events. free survival (PFS), and adverse events.

Reports on cytotoxic anticancer agents for MCC have primarily focused on cases treated before the advent of immune checkpoint inhibitors [36, 37, 38, 39], and no studies or reports have been published on cases resistant to or unsuitable for immunotherapy. Therefore, we analyzed reports and studies on cytotoxic anticancer agents used primarily as first‐line therapy for MCC and used their results as alternative outcomes. Additionally, due to the scarcity of reports, there were no studies comparing treatment groups with EP/EC therapy alone as the control and treatment groups including only chemotherapy other than EP/EC therapy as the intervention under the same treatment line conditions (e.g., first‐line therapy vs. first‐line therapy, second‐line therapy vs. second‐line therapy). Therefore, even if a treatment group included multiple chemotherapy regimens, studies where the control and intervention regimens each accounted for more than half of the treatment group were included in the review. Under these conditions, the search and secondary screening resulted in the inclusion of five retrospective studies.

#### 12.4.1 EP/EC Therapy vs. Other Cytotoxic Anticancer Agents

ORR was evaluated in three studies. In the systematic review by Nghiem et al. [38], regimens containing platinum agents (cisplatin/carboplatin) with or without etoposide were compared to regimens without platinum agents. When platinum‐based regimens were interpreted as an alternative control (C), ORR was higher for regimens containing platinum‐based agents (50% vs. 33.3%). In the retrospective study by Cowey et al. [37], the chemotherapy group (67 patients in total) containing many patients receiving EP/EC therapy (55 patients, 82.1%) and the chemotherapy group containing many patients receiving topotecan and cyclophosphamide + doxorubicin + vincristine (CAV therapy) (12 patients, 60%) were compared, but no significant difference in ORR was observed (29.4% vs. 28.6%, no significance test). A retrospective study by Fenig et al. [36] analyzed cases receiving EP therapy, cyclophosphamide + methotrexate + 5‐FU (CMF therapy), single‐agent etoposide, and CAV therapy. Although single‐agent etoposide showed a higher ORR (EP vs. CMF, etoposide, CAV: 60% vs. 60%, 100%, 50%), the sample sizes were small (10 patients in the EP group, 10 in the CMF group, 4 in the single‐agent etoposide group, and 2 in the CAV group), and no significance test was performed [36].

DOR was evaluated in two studies. In the retrospective study by Cowey et al. [36] described above, the EP/EC chemotherapy group tended to have a longer DOR than the topotecan and CAV therapy groups (median DOR: EP/EC 6.7 months vs. topotecan and CAV 1.7 months, no significance test). In the retrospective study by Fenig et al. [36], the median DOR was 20 months in the CMF group, 3 months in the etoposide monotherapy group, and 2 months in the CAV group, compared with 5 months in the EP group.

In the retrospective study by Cowey et al. [37], OS tended to be longer in the EP/EC‐based chemotherapy group than in the topotecan and CAV‐based chemotherapy group (median OS: EP/EC 10.5 months vs. topotecan and CAV 4.3 months, no significance test). In a retrospective study by Fenig et al. [36], the median OS was 34 months in the CMF group, 19 months in the etoposide monotherapy group, and 17 months in the CAV group, compared with 17 months in the EP group.

PFS was evaluated only in the retrospective study by Cowey et al. [37] and tended to be longer in the EP/EC‐based chemotherapy group than in the topotecan and CAV‐based chemotherapy groups (median PFS: EP/EC 4.6 months vs. topotecan and CAV 2.2 months), no significance test.

Adverse events were also evaluated in only one retrospective study, and the incidence of adverse events leading to treatment discontinuation was not significantly different in the EP/EC‐based chemotherapy group compared with the topotecan and CAV‐based chemotherapy groups (EP/EC 33.3% vs. topotecan and CAV 35.7%, no significance test) [37].

#### 12.4.2 EP/EC Therapy vs. Avelumab

Differences between avelumab and EP/EC therapy were also compared for reference.

For ORR, the avelumab group tended to be higher (avelumab 64.3% vs. chemotherapy 42.5%, no significance test) than the EP/EC therapy‐dominated chemotherapy group (hereafter referred to as the chemotherapy group in this section) [40].

The DOR tended to be longer in the chemotherapy group than in the avelumab group in the same report (median DOR: chemotherapy 44.5 months vs. avelumab 15.5 months, no significance test) [40].

Regarding OS and PFS, the median tended to be longer in the avelumab group than in the chemotherapy group (median OS: avelumab 20.2 months vs. chemotherapy 14.7 months; median PFS: avelumab 11.4 months vs. chemotherapy 6.1 months) [40].

Regarding adverse events, the incidence of adverse events leading to treatment discontinuation was lower in the chemotherapy group than in the avelumab group (chemotherapy 12.5% vs. avelumab 16.7%, no significance test) [40].

Although outside the intervention setting of this CQ, a study comparing no treatment observation versus all systemic chemotherapy showed no significant difference in OS (2‐year survival: no treatment 68% vs. chemotherapy 41%; *p* = 0.222) [39], but the details of the study are unknown because only conference abstracts were available.

### 12.5 Comments

None of the five studies included in the systematic review compared EP/EC with systemic chemotherapy other than EP/EC in second‐line treatment. In addition, only one of the studies was able to compare EP/EC and systemic chemotherapy other than EP/EC, and the number of cases was small, [36]. Therefore, the panel meeting included a comparison of platinum‐containing and non‐platinum‐containing regimens by Nghiem et al. [38], a comparison of the EP/EC‐based chemotherapy group with the topotecan and CAV‐based chemotherapy group by Cowey et al. [37], a comparison of the EP/EC‐based chemotherapy group with the avelumab group as the first‐line therapy [40], and first‐line systemic chemotherapy and no treatment observation [39]. The study by Cowey et al. [37] had a large sample size. However, it featured heterogeneous chemotherapy regimens, with first‐line EP/EC compared to second‐line topotecan and CAV, thereby contrasting different treatment lines. Therefore, it was not possible to identify a clear outcome for this CQ. We concluded that this was not an appropriate comparison for the intervention–control evaluation.

After the above discussion at the panel meeting, the first vote was taken with one member weakly recommending no intervention, and the remaining seven members voting for “unable to determine recommendation”. After another thorough discussion, a second vote was taken, and the result was the same: “No clear recommendation can be determined for any systemic chemotherapy for advanced MCC refractory or unsuitable for immunotherapy”.

### 12.6 Salient Aspects for Clinical Application

Although the efficacy of EP/EC as a second‐line therapy is unknown, it can be assumed that EP/EC will continue to be used as a standard treatment for avelumab‐failure/unsuitable patients in the future due to its extensive experience as a first‐line therapy in actual clinical practice. On the other hand, these guidelines did not provide a clear recommendation because the superiority of other systemic chemotherapy regimens compared to EP/EC is unknown, but the use of these regimens as second‐line therapy should not be ruled out and is acceptable.

While except for cases such as TMB‐high, avelumab is the only systemic therapy covered by insurance for MCC. Therefore, for patients who do not respond to avelumab or who are not suitable for avelumab, EP/EC, topotecan, CAV, or other cytotoxic agents should be considered after careful consideration of age, complications, and compliance with the regulations of the ethics committee of the respective institution. It should also be explained that the patient is not covered by the Adverse Reactions Relief Program, and palliative/supportive care is also an option.

### 12.7 Study Subjects in the Future

Although avelumab, the only chemotherapy covered by insurance, is the first‐line treatment for MCC, there is insufficient evidence to develop a treatment strategy for second‐line treatment and beyond. However, there is insufficient evidence to develop a treatment strategy for second‐line and subsequent therapies. Therefore, it is necessary to investigate the efficacy and safety of avelumab in Japan, as well as the outcomes of cytotoxic anticancer agents, including EP/EC, administered after second‐line therapy after avelumab failure in registry studies and multicenter backward‐looking studies.

## Conflicts of Interest

In accordance with the “Guidance on Eligibility Criteria for Participation in the Formulation of Medical Practice Guidelines” (hereafter referred to as “Guidance on Eligibility Criteria”) [41] published by the Japan Medical Association in June 2023, guidelines development committee members and external evaluation committee members disclosed their conflicts of interest (COI) for the past 3 years back to the previous year and for each year until the guidelines were published when they took office. The disclosure of conflicts of interest (COI) was conducted for the past 3 years retroactively from the previous year and for each year up to the publication of the guidelines. When reporting, the COI of (1) the committee members themselves, (2) their spouses, (3) their first‐degree relatives or those with whom they shared income or property interests, and (4) the COI of the organizations and departments to which they belonged, were reported along with the monetary categories on the COI self‐report form provided in the Participation Standards Guidance. The study period was from January 1, 2020, to December 31, 2023. There were no members of the MCC guidelines development committee or the systematic review team that needed to be declared. The presence or absence of COI was checked again at the time of voting for each CQ. T.M. is an editorial board member of The Journal of Dermatology. To minimize bias, T.M. was excluded from all editorial decision‐making related to the acceptance of this article for publication.

## Supporting information


**Table S1:** The list of committee members involved in the MCC guideline development.

**Table S2:** Results of literature search with relevant keywords in each clinical question.

## Data Availability

Data sharing was not applicable to this article as no datasets were generated or analyzed during this study.

## References

[1] T. Amaral , U. Leiter , and C. Garbe , “Merkel Cell Carcinoma: Epidemiology, Pathogenesis, Diagnosis and Therapy,” Reviews in Endocrine & Metabolic Disorders 18 (2017): 517–532. 28916903 10.1007/s11154-017-9433-0

[2] K. G. Paulson , S. Y. Park , N. A. Vandeven , et al., “Merkel Cell Carcinoma: Current US Incidence and Projected Increases Based on Changing Demographics,” Journal of the American Academy of Dermatology 78 (2018): 457–463.e2. 29102486 10.1016/j.jaad.2017.10.028 PMC5815902

[3] D. Ogata , K. Namikawa , E. Nakano , et al., “Epidemiology of Skin Cancer Based on Japan's National Cancer Registry 2016‐2017,” Cancer Science 114 (2023): 2986–2992. 37095610 10.1111/cas.15823 PMC10323082

[4] M. Nakamura and A. Morita , “Immune Activity in Merkel Cell Carcinoma,” Journal of Dermatology 49 (2022): 68–74. 34766373 10.1111/1346-8138.16232 PMC9299685

[5] K. L. Harms , M. A. Healy , P. Nghiem , et al., “Analysis of Prognostic Factors From 9387 Merkel Cell Carcinoma Cases Forms the Basis for the New 8th Edition AJCC Staging System,” Annals of Surgical Oncology 23 (2016): 3564–3571. 27198511 10.1245/s10434-016-5266-4 PMC8881989

[6] T. Morizane , A. Okumura , Y. Sato , T. Baba , T. Fukuoka , and M. Yoshida , Minds Clinical Practice Guideline Development Manual 2020. Ver. 3.0.

[7] NCCN , “Clinical Practice Guidelines in Oncology (NCCN Guidelines),” (2024). Merkel Cell Carcinoma Version 1.2024.

[8] F. Jaouen , T. Kervarrec , A. Caille , et al., “Narrow Resection Margins Are Not Associated With Mortality or Recurrence in Patients With Merkel Cell Carcinoma: A Retrospective Study,” Journal of the American Academy of Dermatology 84 (2021): 921–929. 33253832 10.1016/j.jaad.2020.11.038

[9] N. Andruska , B. W. Fischer‐Valuck , L. Mahapatra , et al., “Association Between Surgical Margins Larger Than 1 Cm and Overall Survival in Patients With Merkel Cell Carcinoma,” JAMA Dermatology 157 (2021): 540–548. 33760021 10.1001/jamadermatol.2021.0247 PMC7992025

[10] M. C. Perez , F. R. de Pinho , A. Holstein , et al., “Resection Margins in Merkel Cell Carcinoma: Is a 1‐Cm Margin Wide Enough?,” Annals of Surgical Oncology 25 (2018): 3334–3340. 30073600 10.1245/s10434-018-6688-y PMC7771268

[11] E. S. Tarabadkar , T. Fu , K. Lachance , et al., “Narrow Excision Margins Are Appropriate for Merkel Cell Carcinoma When Combined With Adjuvant Radiation: Analysis of 188 Cases of Localized Disease and Proposed Management Algorithm,” Journal of the American Academy of Dermatology 84 (2021): 340–347. 32711093 10.1016/j.jaad.2020.07.079 PMC7855019

[12] N. A. Alexander , S. K. Schaub , P. H. Goff , et al., “Increased Risk of Recurrence and Disease‐Specific Death Following Delayed Postoperative Radiation for Merkel Cell Carcinoma,” Journal of the American Academy of Dermatology 90 (2024): 261–268. 37778663 10.1016/j.jaad.2023.07.1047 PMC11260506

[13] Y. Song , F. S. Azari , R. Tang , et al., “Patterns of Metastasis in Merkel Cell Carcinoma,” Annals of Surgical Oncology 28 (2021): 519–529. 32405979 10.1245/s10434-020-08587-3 PMC7220648

[14] J. G. Iyer , B. E. Storer , K. G. Paulson , et al., “Relationships Among Primary Tumor Size, Number of Involved Nodes, and Survival for 8044 Cases of Merkel Cell Carcinoma,” Journal of the American Academy of Dermatology 70 (2014): 637–643. 24521828 10.1016/j.jaad.2013.11.031 PMC3959572

[15] R. R. Z. Conic , J. Ko , S. Saridakis , et al., “Sentinel Lymph Node Biopsy in Merkel Cell Carcinoma: Predictors of Sentinel Lymph Node Positivity and Association With Overall Survival,” Journal of the American Academy of Dermatology 81 (2019): 364–372. 30902726 10.1016/j.jaad.2019.03.027

[16] Y. G. Karunaratne , D. A. Gunaratne , and M. J. Veness , “Systematic Review of Sentinel Lymph Node Biopsy in Merkel Cell Carcinoma of the Head and Neck,” Head & Neck 40 (2018): 2704–2713. 29934958 10.1002/hed.25345

[17] D. A. Gunaratne , J. R. Howle , and M. J. Veness , “Sentinel Lymph Node Biopsy in Merkel Cell Carcinoma: A 15‐Year Institutional Experience and Statistical Analysis of 721 Reported Cases,” British Journal of Dermatology 174 (2016): 273–281. 26480031 10.1111/bjd.14240

[18] S. D. Kachare , J. H. Wong , N. A. Vohra , E. E. Zervos , and T. L. Fitzgerald , “Sentinel Lymph Node Biopsy Is Associated With Improved Survival in Merkel Cell Carcinoma,” Annals of Surgical Oncology 21 (2014): 1624–1630. 24378985 10.1245/s10434-013-3434-3

[19] M. Yaghi , P. Benedetto , J. Greskovich , et al., “Merkel Cell Carcinoma: Epidemiology, Disease Presentation, and Current Clinical Practice Outcomes,” Journal of the American Academy of Dermatology International 9 (2022): 128–136. 36262427 10.1016/j.jdin.2022.06.004 PMC9574710

[20] I. J. Moon , H. Na , H. S. Cho , et al., “Clinicopathological Characteristics and Prognosis of Merkel Cell Carcinoma: A Single‐Center Retrospective Study in Korea,” Journal of Cancer Research and Clinical Oncology 149 (2023): 10065–10074. 37261524 10.1007/s00432-023-04932-7 PMC11796858

[21] Y. Xia , D. Cao , J. Zhao , B. Z. Zhu , and J. Xie , “Clinical Features and Prognosis of Merkel Cell Carcinoma in Elderly Patients,” Medical Science Monitor 26 (2020): e924570. 32653892 10.12659/MSM.924570 PMC7375029

[22] E. Sattler , T. Geimer , I. Sick , et al., “Sentinel Lymph Node in Merkel Cell Carcinoma: To Biopsy or Not to Biopsy?,” Journal of Dermatology 40 (2013): 374–379. 23414107 10.1111/1346-8138.12072

[23] S. Bhatia , B. E. Storer , J. G. Iyer , et al., “Adjuvant Radiotherapy and Chemotherapy in Merkel Cell Carcinoma: Survival Analyses of 6908 Cases From the National Cancer Data Base,” Journal of the National Cancer Institute 108 (2016): djw042. 27245173 10.1093/jnci/djw042 PMC6059142

[24] J. A. Vargo , E. R. Ghareeb , G. K. Balasubramani , and S. Beriwal , “RE: Adjuvant Radiotherapy and Chemotherapy in Merkel Cell Carcinoma: Survival Analyses of 6908 Cases From the National Cancer Data Base,” Journal of the National Cancer Institute 109 (2017): djx052. 10.1093/jnci/djx052 28423400

[25] B. Singh , M. M. Qureshi , M. T. Truong , and D. Sahni , “Demographics and Outcomes of Stage I and II Merkel Cell Carcinoma Treated With Mohs Micrographic Surgery Compared With Wide Local Excision in the National Cancer Database,” Journal of the American Academy of Dermatology 79 (2018): 126–134. 29408552 10.1016/j.jaad.2018.01.041

[26] S. H. Kang , L. E. Haydu , R. Y. Goh , and G. B. Fogarty , “Radiotherapy Is Associated With Significant Improvement in Local and Regional Control in Merkel Cell Carcinoma,” Radiation Oncology 7 (2012): 171. 23075308 10.1186/1748-717X-7-171 PMC3494567

[27] S. R. Takagishi , T. E. Marx , C. Lewis , et al., “Postoperative Radiotherapy Is Associated With a Reduced Risk of Local Recurrence Among Low Risk Merkel Cell Carcinomas of the Head and Neck,” Advances in Radiation Oncology 1 (2016): 244–251. 28740894 10.1016/j.adro.2016.10.003 PMC5514235

[28] R. J. Straker , M. J. Carr , A. J. Sinnamon , et al., “Predictors of False Negative Sentinel Lymph Node Biopsy in Clinically Localized Merkel Cell Carcinoma,” Annals of Surgical Oncology 28 (2021): 6995–7003. 33890195 10.1245/s10434-021-10031-z

[29] T. E. Grotz , R. W. Joseph , B. A. Pockaj , et al., “Negative Sentinel Lymph Node Biopsy in Merkel Cell Carcinoma Is Associated With a Low Risk of Same‐Nodal‐Basin Recurrences,” Annals of Surgical Oncology 22 (2015): 4060–4066. 25676844 10.1245/s10434-015-4421-7

[30] L. A. Dinges , T. Eichkorn , S. Regnery , et al., “Postoperative Radiotherapy and the Role of Regional Lymph Node Irradiation in Localized Merkel Cell Carcinoma: A Single‐Center Retrospective Analysis,” Cancers 14 (2022): 6140. 36551625 10.3390/cancers14246140 PMC9776017

[31] J. C. Becker , A. J. Beer , V. K. DeTemple , et al., “S2k Guideline ‐ Merkel Cell Carcinoma (MCC, Neuroendocrine Carcinoma of the Skin) ‐ Update 2022,” Journal der Deutschen Dermatologischen Gesellschaft 21 (2023): 305–320. 10.1111/ddg.14930 36929552

[32] K. L. Ma , C. E. Sharon , G. N. Tortorello , et al., “Radiation, Lymph Node Dissection, or Both: Management of Lymph Node Micrometastases From Merkel Cell Carcinoma,” Annals of Surgical Oncology 30 (2023): 4345–4355. 37106277 10.1245/s10434-023-13437-z

[33] M. C. Perez , D. E. Oliver , E. S. Weitman , et al., “Management of Sentinel Lymph Node Metastasis in Merkel Cell Carcinoma: Completion Lymphadenectomy, Radiation, or Both?,” Annals of Surgical Oncology 26 (2019): 379–385. 30311164 10.1245/s10434-018-6810-1 PMC7771265

[34] N. Andruska , L. Mahapatra , R. J. Brenneman , et al., “Regional Lymph Node Irradiation in Locally Advanced Merkel Cell Carcinoma Reduces Regional and Distant Relapse and Improves Disease‐Specific Survival,” Radiotherapy and Oncology 155 (2021): 246–253. 33212121 10.1016/j.radonc.2020.11.003

[35] J. S. Lee , A. B. Durham , C. K. Bichakjian , et al., “Completion Lymph Node Dissection or Radiotherapy for Sentinel Node Metastasis in Merkel Cell Carcinoma,” Annals of Surgical Oncology 26 (2019): 386–394. 30556118 10.1245/s10434-018-7072-7

[36] E. Fenig , B. Brenner , A. Katz , E. Rakovsky , M. B. Hana , and A. Sulkes , “The Role of Radiation Therapy and Chemotherapy in the Treatment of Merkel Cell Carcinoma,” Cancer 80 (1997): 881–885. 9307187 10.1002/(sici)1097-0142(19970901)80:5<881::aid-cncr8>3.0.co;2-o

[37] C. L. Cowey , L. Mahnke , J. Espirito , C. Helwig , D. Oksen , and M. Bharmal , “Real‐World Treatment Outcomes in Patients With Metastatic Merkel Cell Carcinoma Treated With Chemotherapy in the USA,” Future Oncology 13 (2017): 1699–1710. 28605939 10.2217/fon-2017-0187

[38] P. Nghiem , H. L. Kaufman , M. Bharmal , L. Mahnke , H. Phatak , and J. C. Becker , “Systematic Literature Review of Efficacy, Safety and Tolerability Outcomes of Chemotherapy Regimens in Patients With Metastatic Merkel Cell Carcinoma,” Future Oncology 13 (2017): 1263–1279. 28350180 10.2217/fon-2017-0072 PMC6040046

[39] A. J. Sabol , S. Markovic , C. Otley , and K. A. R. Price , “Benefit of Chemotherapy (CT) in Patients (Pts) With Merkel Cell Carcinoma (MCC) With Distant Metastases (Mets) and Visceral Organ Involvement,” Journal of Clinical Oncology 34 (2016): e21000.

[40] A. Bhanegaonkar , F. X. Liu , M. Boyd , et al., “Real‐World Clinical Outcomes in Patients With Locally Advanced or Metastatic Merkel Cell Carcinoma Treated in U.S. Oncology Clinical Practices: Results From SPEAR‐Merkel,” Oncologist 26 (2021): e1633–43. 34101298 10.1002/onco.13845 PMC8417847

[41] Japan Medical Association Committee on Conflicts of Interest , Guidance on Eligibility Criteria for Participation in Clinical Practice Guideline Development (Japan Medical Association, 2017), 1–18.

