# Supplementary material for: Japanese Dermatological Association Guidelines: Clinical Questions of Guidelines for Merkel Cell Carcinoma 2025
Source: J Dermatol. 2025 Oct 8;52(11):e954–67. doi: 10.1111/1346-8138.17974 (PMC12592585; doi:10.1111/1346-8138.17974)
Supplement: Supplementary file 1 — Table S1: The list of committee members involved in the MCC guideline development. [file JDE-52-e954-s002.docx]

Table S1 The list of committee members involved in the MCC guideline development

| Chairman of Executive Committee | Yasuhiro Nakamura (Saitama Medical University International Medical Center) |
| --- | --- |
| Member of Executive Committee | Hiroshi Koga (Kitatoda Alps Dermatology Clinic) |
|  | Hiroshi Uchi (National Hospital Organization Kyushu Cancer Center) |
|  | Tomomitsu Miyagaki (St. Marianna University School of Medicine) |
| Chairman of Committee | Motoki Nakamura (Nagoya City University) |
| Board of Directors (Guideline Development Team) | Kotaro Nagase (Nagase Dermatology Clinic) |
|  | Junji Kato (Sapporo Medical University School of Medicine) |
|  | Masahito Yasuda (Gunma University) |
|  | Natsuo Tomita (Nagoya City University) |
| Board of Directors (Systematic Review Team) | Tadahiro Kobayashi (Public Central Hospital of Matto Ishikawa) |
|  | Keitaro Fukuda (Keio University) |
|  | Akihiko Yuki (Niigata University) |
