# Supplementary material for: Japanese Dermatological Association Guidelines: Clinical Questions of Guidelines for Merkel Cell Carcinoma 2025
Source: J Dermatol. 2025 Oct 8;52(11):e954–67. doi: 10.1111/1346-8138.17974 (PMC12592585; doi:10.1111/1346-8138.17974)
Supplement: Supplementary file 2 — Table S2: Results of literature search with relevant keywords in each clinical question. [file JDE-52-e954-s001.docx]

**Table S2. Results of literature search with relevant keywords in each clinical question**

| CQ1 | Is a lateral margin of less than 1 cm recommended during surgical treatment of primary Merkel cell carcinoma? |
| --- | --- |
| database | The Cochrane Library (CDSR, CCRCT), PubMed, Medical Central Journal |

[The Cochrane Library]

| ♯ | search expression | number of documents |
| --- | --- | --- |
| #01 | ([mh "Carcinoma, Merkel Cell"] OR "merkel cell" NEXT (carcinoma* OR cancer* OR tumor* OR tumour*):ti,ab,kw | 108 |
| #02 | ([mh "Neoplasms, Multiple Primary"] OR [mh "Neoplasms, Unknown Primary"] OR primary:ti,ab,kw) | 558684 |
| #03 | ([mh "Dermatologic Surgical Procedures"] OR (excision* OR resection* OR surg* OR exeresis OR operation*):ti,ab,kw) | 341425 |
| #04 | [mh "Margins of Excision"] OR margin*:ti,ab,kw | 24756 |
| #05 | #1 and #2 and #3 and #4 | 3 |

[PubMed].

| ♯ | search expression | number of documents |
| --- | --- | --- |
| #01 | ("carcinoma, merkel cell"[MeSH Terms] OR "merkel cell carcinoma*" OR "merkel cell cancer*" OR "merkel cell tumor*" OR "merkel cell tumour*") | 4471 |
| #02 | ("neoplasms, multiple primary"[MeSH Terms] OR "neoplasms, unknown primary"[MeSH Terms] OR "primary") | 2028746 |
| #03 | ("dermatologic surgical procedures"[MeSH Terms] OR "surgery"[MeSH Subheading] OR "excision*" OR "resection*" OR "surgery*" OR "exeresis" OR " operation*") | 4180352 |
| #04 | ("Margins of Excision"[MeSH Terms] OR "margin*"[Text Word]) AND 1972/01/01:2023/06/30[Date - Publication]. | 245592 |
| #05 | #1 and #2 and #3 and #4 | 109 |

[Central Journal of Medicine]

| ♯ | search expression | number of documents |
| --- | --- | --- |
| #01 | (Merkel cell carcinoma/TH or Merkel cell carcinoma/AL) | 1667 |
| #02 | (tumor - multiple primary/TH or tumor - unknown primary/TH or primary/AL) | 218461 |
| #03 | (Dermatologic surgery/TH or resection/AL or surgery/AL or surgery/AL or SH=surgical therapy) or (excised tissue margins/TH or margins/AL) | 3798611 |
| #04 | DT=1972:2023 | 13770235 |
| #05 | #1 and #2 and #3 and #4 | 124 |

| CQ2 | Is sentinel node biopsy recommended for Merkel cell carcinoma? |
| --- | --- |
| database | The Cochrane Library (CDSR, CCRCT), PubMed, Medical Central Journal |

[The Cochrane Library]

| ♯ | search expression | number of documents |
| --- | --- | --- |
| #01 | ([mh "Carcinoma, Merkel Cell"] OR   ("merkel cell" NEXT (carcinoma* OR cancer* OR tumor* OR tumour*)):ti,ab,kw) | 108 |
| #02 | ([mh "Sentinel Lymph Node Biopsy"] OR "Sentinel Lymph Node":ti,ab,kw) | 1,416 |
| #03 | #1 AND #2 with Publication Year from 1972 to 2023, in Trials | 8 |

[PubMed].

| ♯ | search expression | number of documents |
| --- | --- | --- |
| #01 | "carcinoma"[Title] OR "cancer"[Title] OR "tumor"[Title] OR "tumour"[Title]. | 1,936,694 |
| #02 | merkel cell[Title]. | 3,534 |
| #03 | #1 AND #2 | 3,063 |
| #04 | "carcinoma, merkel cell" [MeSH Terms]. | 3,170 |
| #05 | #3 OR #4 | 3,760 |
| #06 | "Sentinel Lymph Node Biopsy" [MeSH Terms]. | 13,442 |
| #07 | "Sentinel Lymph Node"[Title]. | 6,162 |
| #08 | #6 OR #7 | 14,820 |
| #09 | 1972/01/01:2023/06/30[Date - Publication]. | 32,187,145 |
| #10 | #5 AND #8 AND #9 | 203 |

[Central Journal of Medicine].

| ♯ | search expression | number of documents |
| --- | --- | --- |
| #01 | Merkel cell carcinoma/TH | 1,611 |
| #02 | Merkel cell carcinoma/AL | 958 |
| #03 | #1 OR #2 | 1,677 |
| #04 | Sentinel node biopsy/TH | 7,848 |
| #05 | Sentinel lymph node/AL | 12,578 |
| #06 | #4 OR #5 | 12,578 |
| #07 | #3 AND #6 AND (DT=1972:2023) | 83 |

| CQ3-1 | Is postoperative radiotherapy to the primary tumor recommended for the edge-negative case after resection of the primary Merkel cell carcinoma? |
| --- | --- |
| database | The Cochrane Library, PubMed, Central Journal of Medicine |

[The Cochrane Library]

| ♯ | search expression | number of documents |
| --- | --- | --- |
| #01 | ([mh "Carcinoma, Merkel Cell"] OR ("merkel cell" NEXT (carcinoma* OR cancer* OR tumor* OR tumour*)):ti,ab,kw) | 108 |
| #02 | ([mh "Radiotherapy, Adjuvant"] OR (adjuvant NEXT (radiotherap* OR radiation* OR RT)):ti,ab,kw OR (postoperative NEXT (radiotherap* OR radiation *)):ti,ab,kw) | 3,880 |
| #03 | ([mh "Neoplasms, Multiple Primary"] OR [mh "Neoplasms, Unknown Primary"] OR primary:ti,ab,kw) | 558,685 |
| #04 | ((surg* OR excision* OR resection* OR operation*):ti,ab,kw) | 341,101 |
| #05 | #1 and #2 and #3 and #4 with Publication Year from 1972 to 2023, in Trials | 10 |

[PubMed].

| ♯ | search expression | number of documents |
| --- | --- | --- |
| #01 | "carcinoma, merkel cell"[MeSH Terms] OR "merkel cell carcinoma*"[Text Word] OR "merkel cell cancer*"[Text Word] OR "merkel cell tumor*"[Text Word] OR "merkel cell tumour*"[Text Word] OR | 4,454 |
| #02 | "radiotherapy, adjuvant"[MeSH Terms] OR "adjuvant radiotherap*"[Text Word] OR "adjuvant radiation*"[Text Word] OR "adjuvant RT"[Text Word] OR " postoperative radiotherap*"[Text Word] OR "postoperative radiation*"[Text Word] OR "radiotherapy"[MeSH Subheading]. | 234,060 |
| #03 | "primary"[Text Word] OR "neoplasms, multiple primary"[MeSH Terms] OR "neoplasms, unknown primary"[MeSH Terms] OR "margin negative"[Text Word]. | 2,021,465 |
| #04 | "surgery*"[Text Word] OR "excision*"[Text Word] OR "resection*"[Text Word] OR "operation*"[Text Word] OR "surgery"[MeSH Subheading]. | 4,153,301 |
| #05 | 1972/01/01:2023/06/30[Date - Publication]. | 32,182,495 |
| #06 | #1 and #2 and #3 and #4 and #5 | 225 |

[Medical Central Journal].

| **♯** | **search expression** | **number of documents** |
| --- | --- | --- |
| #01 | Merkel cell carcinoma/TH or Merkel cell carcinoma/AL | 1,670 |
| #02 | Adjuvant Radiation Therapy / TH or Postoperative Radiation / AL or Postoperative Radiation / AL or SH = Radiation Therapy | 15,297 |
| #03 | Tumor - multiple primary/TH or Tumor - unknown primary/TH or primary/AL | 217,894 |
| #04 | Surgery/AL or resection/AL or surgery/AL or SH = surgical therapy | 3,781,498 |
| #05 | #1 and #2 and #3 and #4 | 23 |
| #06 | DT=1972:2023 | 13,734,846 |
| #07 | #5 and #6 | 23 |

| CQ3-2 | If sentinel node metastasis is negative, is postoperative radiation therapy to the regional lymph node group recommended? |
| --- | --- |
| database | The Cochrane Library, PubMed, Central Journal of Medicine |

[The Cochrane Library]

| ♯ | search expression | number of documents |
| --- | --- | --- |
| #01 | ([mh "Carcinoma, Merkel Cell"] OR ("merkel cell" NEXT (carcinoma* OR cancer* OR tumor* OR tumour*)):ti,ab,kw) | 108 |
| #02 | ([mh "Radiotherapy, Adjuvant"] OR (adjuvant NEXT (radiotherap* OR radiation* OR RT)):ti,ab,kw OR (postoperative NEXT (radiotherap* OR radiation *)):ti,ab,kw) | 3,880 |
| #03 | ([mh "Sentinel Lymph Node Biopsy"] OR [mh "Sentinel Lymph Node"] OR ("sentinel lymph node"):ti,ab,kw) | 1,416 |
| #04 | #1 and #2 an #3 with Publication Year from 1972 to 2023, in Trials | 4 |

[PubMed].

| ♯ | search expression | number of documents |
| --- | --- | --- |
| #01 | "carcinoma, merkel cell"[MeSH Terms] OR "merkel cell carcinoma*"[Text Word] OR "merkel cell cancer*"[Text Word] OR "merkel cell tumor*"[Text Word] OR "merkel cell tumour*"[Text Word] OR | 4,455 |
| #02 | "radiotherapy, adjuvant"[MeSH Terms] OR "adjuvant radiotherap*"[Text Word] OR "adjuvant radiation*"[Text Word] OR "adjuvant RT"[Text Word] OR " postoperative radiotherap*"[Text Word] OR "postoperative radiation*"[Text Word] OR "radiotherapy"[MeSH Subheading]. | 234,061 |
| #03 | "sentinel lymph node biopsy"[MeSH Terms] OR "Sentinel Lymph Node"[MeSH Terms] OR "Sentinel Lymph Node"[Text Word]. | 18,073 |
| #04 | 1972/01/01:2023/06/30[Date - Publication]. | 32,183,187 |
| #05 | #1 and #2 and #3 and #4 | 99 |

[Central Journal of Medicine].

| **♯** | **search expression** | **number of documents** |
| --- | --- | --- |
| #01 | Merkel cell carcinoma/TH or Merkel cell carcinoma/AL | 1,670 |
| #02 | Adjuvant Radiation Therapy / TH or Postoperative Radiation / AL or Postoperative Radiation / AL or SH = Radiation Therapy | 15,297 |
| #03 | Sentinel node biopsy/TH or sentinel node/TH or sentinel node metastasis/AL | 11,044 |
| #04 | #1 and #2 and #3 | 15 |
| #05 | DT=1972:2023 | 13,734,846 |
| #06 | #4 and #5 | 15 |

| CQ3-3 | Is postoperative radiation therapy to regional lymph node groups recommended after regional lymph node dissection? |
| --- | --- |
| database | The Cochrane Library, PubMed, Central Journal of Medicine |

[The Cochrane Library]

| ♯ | search expression | number of documents |
| --- | --- | --- |
| #01 | ([mh "Carcinoma, Merkel Cell"] OR ("merkel cell" NEXT (carcinoma* OR cancer* OR tumor* OR tumour*)):ti,ab,kw) | 108 |
| #02 | ([mh "Radiotherapy, Adjuvant"] OR (adjuvant NEXT (radiotherap* OR radiation* OR RT)):ti,ab,kw OR (postoperative NEXT (radiotherap* OR radiation *)):ti,ab,kw) | 3,880 |
| #03 | ([mh "Sentinel Lymph Node Biopsy"] OR [mh "Sentinel Lymph Node"] OR ("sentinel lymph node"):ti,ab,kw) | 1,416 |
| #04 | #1 and #2 an #3 with Publication Year from 1972 to 2023, in Trials | 4 |

[PubMed].

| ♯ | search expression | number of documents |
| --- | --- | --- |
| #01 | "carcinoma, merkel cell"[MeSH Terms] OR "merkel cell carcinoma*"[Text Word] OR "merkel cell cancer*"[Text Word] OR "merkel cell tumor*"[Text Word] OR "merkel cell tumour*"[Text Word] OR | 4,455 |
| #02 | "radiotherapy, adjuvant"[MeSH Terms] OR "adjuvant radiotherap*"[Text Word] OR "adjuvant radiation*"[Text Word] OR "adjuvant RT"[Text Word] OR " postoperative radiotherap*"[Text Word] OR "postoperative radiation*"[Text Word] OR "radiotherapy"[MeSH Subheading]. | 234,061 |
| #03 | "sentinel lymph node biopsy"[MeSH Terms] OR "Sentinel Lymph Node"[MeSH Terms] OR "Sentinel Lymph Node"[Text Word]. | 18,073 |
| #04 | 1972/01/01:2023/06/30[Date - Publication]. | 32,183,187 |
| #05 | #1 and #2 and #3 and #4 | 99 |

[Central Journal of Medicine].

| **♯** | **search expression** | **number of documents** |
| --- | --- | --- |
| #01 | Merkel cell carcinoma/TH or Merkel cell carcinoma/AL | 1,670 |
| #02 | Adjuvant Radiation Therapy / TH or Postoperative Radiation / AL or Postoperative Radiation / AL or SH = Radiation Therapy | 15,297 |
| #03 | Sentinel node biopsy/TH or sentinel node/TH or sentinel node metastasis/AL | 11,044 |
| #04 | #1 and #2 and #3 | 15 |
| #05 | DT=1972:2023 | 13,734,846 |
| #06 | #4 and #5 | 15 |

| CQ4 | Is systemic chemotherapy other than EP/EC recommended for advanced stage Merkel cell carcinoma that is refractory or unsuitable for immunotherapy? |
| --- | --- |
| database | The Cochrane Library (CDSR, CCRCT), PubMed, Medical Central Journal |

[The Cochrane Library]

| ♯ | search expression | number of documents |
| --- | --- | --- |
| #1 | ([mh "Carcinoma, Merkel Cell"] OR ("merkel cell" NEXT (carcinoma* OR cancer* OR tumor* OR tumour*))):ti,ab,kw | 107 |
| #2 | ((systemic AND (chemotherap* OR pharmacotherap*)) OR ("systemic drug" NEXT therap*)):ti,ab,kw | 6610 |
| #3 | ([mh "Antineoplastic Combined Chemotherapy Protocols"] OR [mh "Immune Checkpoint Inhibitors"] OR [mh "Immune Checkpoint Inhibitors"]) | 18703 |
| #4 | ([mh "Neoplasm Metastasis"] OR (metasta* OR unresectable OR immunocompetent OR "immunotherapy failure" OR advanced)):ti,ab,kw | 106306 |
| #5 | #2 or #3 | 24279 |
| #6 | #1 and #5 and #4 with Publication Year from 1972 to 2023, in Trials | 11 |

[PubMed].

| ♯ | search expression | number of documents |
| --- | --- | --- |
| #01 | "carcinoma, merkel cell/drug therapy" [MeSH Terms]. | 358 |
| #02 | "merkel cell carcinoma*"[Text Word] OR "merkel cell cancer*"[Text Word] OR "merkel cell tumor*"[Text Word] OR "merkel cell tumour*"[Text Word]. | 4187 |
| #03 | "systemic chemotherap*"[Text Word] OR "systemic drug therap*"[Text Word] OR "systemic pharmacotherap*"[Text Word] OR "Antineoplastic Combined Chemotherapy Protocols"[MeSH Terms] OR "Immune Checkpoint Inhibitors"[Pharmacological Action] OR "Immune Checkpoint Inhibitors"[MeSH Terms] | 201248 |
| #04 | "Neoplasm Metastasis"[MeSH Terms] OR "metasta*"[Text Word] OR "unresectable"[Text Word] OR "immunocompetent"[Text Word] OR "immunotherapy failure"[Text Word] OR "advanced"[Text Word] | 1238735 |
| #05 | 1972/01/01:2023/06/30[Date - Publication]. | 3 |
| #06 | #2 and #3 | 206 |
| #07 | #1 or #6 | 456 |
| #08 | #7 and #4 and #5 | 284 |

[Central Journal of Medicine].

| ♯ | search expression | number of documents |
| --- | --- | --- |
| #1 | Merkel cell carcinoma/TH or Merkel cell carcinoma/AL | 1672 |
| #2 | Drug Therapy/TH or Immune Checkpoint Inhibitors/TH or Systemic Therapy/AL or Chemotherapy/AL | 892518 |
| #3 | Tumor metastasis/TH or metastasis/AL or progression/AL or unresectable/AL | 528235 |
| #4 | (#1 and #2 and #3) and (DT=1972:2023) | 102 |
